# Changes in the expression of the human adenine nucleotide translocase isoforms condition cellular metabolic/proliferative status

**DOI:** 10.1098/rsob.150108

**Published:** 2016-02-03

**Authors:** Aleix Gavaldà-Navarro, Teresa Mampel, Octavi Viñas

**Affiliations:** Departament de Bioquímica i Biologia Molecular, Facultat de Biologia, Institut de Biomedicina de la Universitat de Barcelona, Universitat de Barcelona and CIBER Fisiopatología de la Obesidad y Nutrición, Barcelona, Spain

**Keywords:** adenine nucleotide translocase, cell proliferation, gene expression, glycolysis, cell stress response, mitochondria

## Abstract

Human cells express four mitochondrial adenine nucleotide translocase (hANT) isoforms that are tissue-specific and developmentally regulated. hANT1 is mainly expressed in terminally differentiated muscle cells; hANT2 is growth-regulated and is upregulated in highly glycolytic and proliferative cells; and hANT3 is considered to be ubiquitous and non-specifically regulated. Here, we studied how the expression of hANT isoforms is regulated by proliferation and in response to metabolic stimuli, and examined the metabolic consequences of their silencing and overexpression. In HeLa and HepG2 cells, expression of hANT3 was upregulated by shifting metabolism towards oxidation or by slowed growth associated with contact inhibition or growth-factor deprivation, indicating that hANT3 expression is highly regulated. Under these conditions, changes in hANT2 mRNA expression were not observed in either HeLa or HepG2 cells, whereas in SGBS preadipocytes (which, unlike HeLa and HepG2 cells, are growth-arrest-sensitive cells), hANT2 mRNA levels decreased. Additionally, overexpression of hANT2 promoted cell growth and glycolysis, whereas silencing of hANT3 decreased cellular ATP levels, limited cell growth and induced a stress-like response. Thus, cancer cells require both hANT2 and hANT3, depending on their proliferation status: hANT2 when proliferation rates are high, and hANT3 when proliferation slows.

## 1. Background

Adenine nucleotide translocase (ANT) is an integral inner mitochondrial membrane protein that counter-exchanges newly synthesized ATP in the mitochondrial matrix for cytosolic ADP, allowing the proper function of oxidative phosphorylation (OXPHOS; for a review, see [1]). In addition to this classical transport function, several lines of evidence indicate that ANT is also involved in other cellular activities. For example, ANT plays a role in mitochondrial proton leakage [2] and has long been proposed to participate directly in the mitochondrial transition pore [3], although this role now seems to be more indirect [4,5].

Several genes encode ANT isoforms in the vast majority of organisms, from yeast to humans. In humans, there are four ANT isoforms that exhibit cell- and tissue-specific expression [6–8]. Human ANT1 (hANT1) is prominently expressed in highly oxidative cells of muscle lineage, such as skeletal muscle and heart, but is also expressed in other highly oxidative tissues, such as the brain. hANT1 is the isoform that best fits the classical ANT function in the OXPHOS process because it is in these organs (heart, skeletal muscle and brain) that OXPHOS is most active [9]. The hANT2 isoform is generally expressed at low levels except in tissues with high regenerative activity, such as liver and highly proliferative blood cells [10]; importantly, it is upregulated in many cancer cells [11,12]. hANT2 was first cloned in human fibroblasts as an early response gene [10], and it has been described as a marker of cell proliferation [13]. These characteristics, taken together with the observation that hANT2 is upregulated when OXPHOS activity is generally low, suggest that this isoform may serve functions other than a classic OXPHOS function. Accordingly, it has been proposed that hANT2 is responsible for mitochondrial ATP uptake [14], working in a reverse mode to fuel the multiple mitochondrial energy-dependent activities that this organelle performs. This process is especially relevant in highly glycolytic cells, such as proliferative cancer cells. The hANT3 isoform is ubiquitously expressed. As such, its gene expression has not been a focus of research, although some attempts have been made to elucidate its regulation. In this context, we have reported that hANT3 expression, as well as that of the other isoforms, is regulated by PGC-1α (peroxisome proliferative activated receptor-γ coactivator 1α) through different transcription factors [15]. hANT3 is predicted to serve a classic OXPHOS function in tissues other than heart and skeletal muscle, where the hANT1 isoform represents the major fraction of total translocase activity [16]. Finally, expression of the hANT4 isoform is restricted to male germinal cells, although residual expression of ANT4 has been detected in other tissues, such as liver [7].

A close relationship has also been established between individual human ANT isoforms and the regulation of apoptosis. Accordingly, it has been reported that hANT1 overexpression in cultured cells induces apoptosis predominantly through the intrinsic pathway [17,18]. As hANT2 is prominently associated with proliferative cells, this isoform has been considered anti-apoptotic. In support of this, knockdown of hANT2 sensitizes cells to lonidamine-induced apoptosis [12], and hANT2 knockout induces apoptosis and inhibits tumour growth, both *in vitro* and *in vivo* [19]. Finally, as is the case for hANT1, we have described that hANT3 overexpression induces apoptosis *in vitro* through the regulation of mPTP (mitochondrial permeability transition pore) activity [20].

Although it seems well established in the literature that expression of the hANT2 gene is highly regulated, whereas the hANT3 gene is ubiquitously expressed, our studies on cells in culture suggest a more nuanced view of the regulation of these isoforms. Because the expression of hANT isoforms seems to be particularly sensitive to the metabolic and proliferative status of cells, we have undertaken an extensive study of the differential mRNA expression of hANT1–3 isoforms under various proliferative conditions and in response to different metabolic stimuli in human cell lines. In an attempt to discern the functions of specific hANT isoforms, we have also investigated the effects of overexpression and silencing of hANT2 and hANT3 on cell growth and metabolism. Our results clearly demonstrate that hANT3 is the main isoform regulated by proliferative and metabolic stimuli in HeLa and HepG2 cells, cell lines characterized by not being fully susceptible to growth arrest (i.e. in response to growth-factor deprivation or cell contact). hANT3 is also essential for cell growth, and its silencing results in energy impairment and a cell stress-like response. On the other hand, hANT2 by itself is able to induce cell proliferation and shift cell metabolism towards glycolysis. Thus, both hANT2 and hANT3 are essential for cancer cells.

## 2. Methods

### 2.1. Cell culture

Human HeLa and HepG2 cells were cultured in maintenance medium composed of Dulbecco's Modified Eagle Medium (DMEM) supplemented with 10% (v/v) fetal bovine serum (FBS) and 100 units ml^−1^ penicillin/streptomycin (P/S) (all from Gibco/Life Technologies, Foster City, CA, USA) at 37°C in a humidified 95% air/5% CO_2_ incubator. Human SGBS preadipocytes were grown in Medium A consisting of DMEM containing 10% FBS, 1% P/S, 33 mM biotin, and 17 mM pantothenic acid (Sigma-Aldrich) at 37°C in a humidified 95% air/5% CO_2_ incubator. For proliferation studies, cells were plated in 6-well plates at low density (LD; 5 × 10^4^ cells well^−1^) or high density (HD; 5 × 10^5^ cells well^−1^). Medium was changed every 24 h. HeLa cells plated at LD were treated with rapamycin (20 or 100 nM, as indicated; Sigma-Aldrich, St Louis, MO, USA) or DMSO (vehicle) for 24 h.

### 2.2. Reagents

Dimethyl sulfoxide (DMSO), oligomycin, *N*-acetylcysteine (NAC), antimycin A, lonidamine and staurosporine were obtained from Sigma-Aldrich.

### 2.3. Transient transfection with shRNA

shRNA for specific knockdown of hANT2 (shANT2, 5′-CTGTTGCCGGGTTGACTTCC-3′) and hANT3 (shANT3, 5′-CGCGACCTCCCTCTGCTTCG-3′), and scrambled control shRNA (shANTm, 5′-CGGATCGCTACAAATAAG-3′), were constructed using a BLOCK-iT U6 RNAi Entry Vector Kit (Invitrogen/Life Technologies, Foster City, CA, USA). For transient transfections, cells were plated in 6-well plates and then transfected 24 h later using the FuGENE HD Transfection Reagent (Promega, Madison, WI, USA) as described by the manufacturer. Briefly, the required amount of plasmid was mixed with FuGENE and Opti-MEM (Invitrogen/Life Technologies), and the mix was dispensed into cell media and incubated with cells for 48 h. A transfection efficiency greater than 80% was routinely achieved.

### 2.4. Adenoviral transduction

After plating cells in 6-well plates and inducing differentiation if required, cells were transduced at different multiplicities of infection (MOIs) for 48 h with distinct adenoviruses containing expression constructs for GFP (transduction control), hANT2 or hANT3. Adenoviruses were obtained from the Center for Animal Biotechnology and Gene Therapy (CBATEG, Universitat Autònoma de Barcelona, Bellaterra, Spain). Adenovirus transduction was carried out by mixing the volume of adenovirus needed with 0.5 ml well^–1^ of DMEM containing 0.5 µg ml^–1^ poly-l-lysine (Sigma-Aldrich), added to enhance the efficiency of transduction. After removing maintenance or differentiation media and washing cells with 1× phosphate-buffered saline (PBS), the adenovirus mix was added into each well and plates were incubated for 5 h at 37°C in a humidified 95% air/5% CO_2_ incubator with regular rocking. Thereafter, wells were filled with regular media (2 ml well^−1^) appropriate for each cell line.

### 2.5. RNA isolation, cDNA synthesis and quantitative real-time PCR

Total RNA from cultured cells was isolated using a column-affinity-based method (NucleoSpin RNA II; Macherey-Nagel, Düren, Germany). cDNA was synthesized from 500 ng of total RNA using Multiscribe reverse transcriptase and random-hexamer primers (TaqMan Reverse Transcription Reagents from Applied Biosystems/Life Technologies, Foster City, CA, USA). For mRNA expression analyses, TaqMan quantitative real-time PCR (qPCR) was performed on a 7500 Real-Time PCR System (Applied Biosystems) in a final volume of 20 ml using Platinum Quantitative PCR SuperMix-UDG with ROX reagents (Invitrogen/Life Technologies) and the following primer pair/probe sets specific for the indicated human targets: CCL2 (MCP1), Hs00234140_m1; DDIT3 (CHOP10), Hs00358796_g1; HSPA5, Hs00607129_gH; IL6, Hs00174131_m1; PDK4, Hs00176875_m1; SLC25A4 (hANT1), Hs00154037_m1; SLC25A5 (hANT2), Hs00854499_g1; SLC25A6 (hANT3), Hs00745067_s1; tumour necrosis factor-*α* (TNF-*α*), Hs00174128_m1; and 18S rRNA, Hs99999901_s1 (TaqMan Gene Expression Assays; Applied Biosystems). The relative levels of target mRNA expression were normalized to that of 18S rRNA, used as an endogenous control.

### 2.6. DNA isolation and quantification of mtDNA content

Total DNA was isolated from cultured cells by phenol/chloroform extraction. Mitochondrial DNA (mtDNA) was quantified by real-time PCR amplification of 100 ng of total DNA using a cytochrome *b* (MT-CYTB) primer/probe set (Hs02596867_s1). The results were expressed relative to the quantity of nuclear DNA, which was determined by amplification of the intronless gene CEBP*α* (Hs00269972_s1).

### 2.7. Analysis of proliferation by sulforhodamine B colorimetric assay

Cell density was determined by measuring cellular protein content using the sulforhodamine B (SRB) colorimetric assay [21]. At the indicated times, cells were washed with PBS, fixed with 10% (w/v) trichloroacetic acid for 1 h at 4°C, and stained with 0.4% (w/v) SRB in 1% (v/v) acetic acid for 20 min. After removing excess dye by washing several times with 1% (v/v) acetic acid, stained protein was dissolved in 10 mM Tris-based solution for spectrophotometric determination at 550 nm.

### 2.8. Analysis of proliferation by cell counting

Cell counting was used as an alternative method for determining cell proliferation. At the indicated times, cells were washed with PBS, detached from culture plates by incubating with 200 ml well^−1^ of 0.05% trypsin-EDTA (Gibco) at 37°C for 2 min, and collected in 2 ml well^−1^ of DMEM. After exclusion staining with 0.4% Trypan Blue (Gibco), cells were counted using a Countess Automated Cell Counter platform (Invitrogen).

### 2.9. Analysis of proliferation by DNA content

Total DNA from cultured cells was isolated using a phenol/chloroform extraction method, and DNA concentration was measured using a NanoDrop ND-1000 Spectrophotometer (NanoDrop Technologies, Wilmington, DE, USA).

### 2.10. Determination of glucose and lactate in cell culture medium

Glucose and lactate concentrations at each plating density were quantified in cell culture medium collected every 24 h. Glucose concentration was determined spectrophotometrically at 340 nm using the Glucose Assay Reagent (Sigma-Aldrich). Lactate concentration determination is based on the l-lactate-to-pyruvate transformation mediated by l-lactate dehydrogenase (Roche Diagnostics GmbH, Mannheim, Germany) in the presence of NAD^+^, with subsequent spectrophotometric quantification of the NADH co-product at 340 nm. Both glucose consumption and lactate production are expressed as the difference in the concentration in medium every 24 h normalized to cellular protein content, determined by SRB assay at the corresponding time point.

### 2.11. Determination of d-glucose oxidation

Glucose oxidation was measured using radiolabelled [^14^C(U)]-d-glucose (Hartmann Analytic GmbH, Braunschweig, Germany) as described by the manufacturer. Briefly, cells plated at the indicated densities were grown for 48 h and incubated with DMEM (without d-glucose) supplemented with 3 mM d-glucose and 1.5 mC_i_ ml^−1^ [^14^C(U)]-d-glucose for 3 h at 37°C in a 5% (v/v) CO_2_ atmosphere. Labelled ^14^CO_2_ was then released from the medium by acidification with 3 M HClO_4_ and retained in a CO_2_ trap consisting of Whatman 3 MM Chr paper (Whatman, GE Healthcare, Little Chalfont, UK) impregnated with B-phenylethylamine (Sigma) and positioned over the wells inside the sealed plates. After 1 h, CO_2_ traps were placed in scintillation vials containing 10 ml of scintillation fluid, and the samples were counted using a Packard 2100TR TriCarb Liquid Scintillation Counter (Packard Instrument Company Inc., Meriden, CT). Decays per minute were normalized to cellular protein content determined by SRB assay.

### 2.12. Quantification of cellular ATP

Cells were washed with PBS, and cellular ATP content was determined using an ATP Bioluminescence Assay Kit (HS II; Roche Diagnostics GmbH). Bioluminescence was measured using a Glomax luminometer (Promega). ATP values were expressed relative to total protein concentration, previously quantified in cell lysates using the Bradford method (Bio-Rad Protein Assay; Bio-Rad Laboratories GmbH, Munich, Germany).

### 2.13. Quantification of cytokines in cell culture medium

TNF-*α*, IL6 and MCP-1 protein levels accumulated in HeLa cell culture media 48 h after transfection with shANTm, shANT2 or shANT3 were measured using the MILLIPLEX MAP kit HADCYT-61 K, based on Luminex xMAP multiplexing technology (Millipore Corporation, Billerica, MA, USA).

### 2.14. Protein extraction and Western blotting

Total cell homogenates were prepared by scraping cells in 100 µl well^−1^ of homogenizing buffer (20 mM Na-HEPES pH 7.4, 2.5 mM NaCl, 1 mM EDTA, 40 mM β-glycerophosphate, 2 mM Na_3_VO_4_, 1% NP-40, 1 mM PMSF, 1 mM DTT, one tablet complete protease inhibitor cocktail per 10 ml of buffer; Roche Applied Science), and incubated at 4°C with agitation for 45 min. Lysates were centrifuged at 12 000*g* at 4°C for 5 min to remove membrane fragments. Supernatant protein concentration was quantified using the Bradford method (Bio-Rad Protein Assay). Mitochondria-enriched and cytosolic protein fractions were prepared by collecting cells in 100 µl well^−1^ of homogenizing buffer (250 mM sucrose, 20 mM Na-HEPES pH 7.4, 10 mM KCl, 1.5 mM MgCl_2_, 1 mM EDTA, 1 mM EGTA, 40 mM β-glycerophosphate, 2 mM Na_3_VO_4_, 1 mM PMSF, 1 mM DTT, one tablet complete protease inhibitor cocktail per 10 ml of buffer; Roche Applied Science) and homogenized in an ice-cold Dounce-type glass homogenizer (50 strokes). Lysates were centrifuged at 1500*g* for 5 min at 4°C to remove intact cells, and supernatants were centrifuged again at 16 000*g* for 20 min at 4°C to obtain a cytosolic fraction (supernatant) and a mitochondria-enriched fraction (pellet). Protein was quantified using the Bradford method (Bio-Rad Protein Assay). For Western blotting, 40 µg of protein was heated for 5 min at 95°C under reducing conditions and then resolved by SDS-PAGE on 12% gels for 3 h at a constant voltage of 100 mV. Proteins were transferred electrophoretically to PVDF membranes (Immobilon; Millipore, Billerica, MA, USA) for 1 h at a constant amperage of 400 mA, and then blocked in 1× PBS containing 5% (wt/vol) non-fat milk and 1% Tween-20 (Sigma-Aldrich) for 1 h at room temperature with agitation. Afterwards, membranes were incubated with primary antibodies against ANT (N-19) (Santa Cruz Biotechnology, Santa Cruz, CA, USA), OPA-1 (Abcam, Cambridge, UK), *α*-tubulin (Sigma-Aldrich) or VDAC (Calbiochem, La Jolla, CA, USA) diluted in 1× PBS containing 3% (wt/vol) non-fat milk and 0.1% Tween-20 overnight at 4°C with agitation. After incubation with horseradish peroxidase (HRP)-coupled secondary antibody (diluted in 1× PBS containing 3% (wt/vol) non-fat milk and 0.1% Tween-20) for 1 h at room temperature with agitation, immunoreactive proteins were detected using a chemiluminescent HRP substrate (Millipore) and a Luminescent Image Analyzer LAS-3000 (Fujifilm Life Science, Tokyo, Japan). Signals were quantified using Multi Gauge software (Fujifilm).

### 2.15. Quantification of reactive oxygen species

Reactive oxygen species (ROS) levels were quantified by fluorometry using an OxiSelect Intracellular ROS Assay Kit (Green Fluorescence) (Cell Biolabs Inc., San Diego, CA, USA). Briefly, after removing media and washing with 1× PBS, cells were incubated with 0.1× 2,7-dichlorodihydrofluorescein diacetate (DCFH-DA) in DMEM at 37°C for 1 h. Labelling medium was removed and cells were washed and lysed with 1× Cell Lysis Buffer (provided with the kit) for 5 min with agitation. The fluorogenic activity of DCF (generated by the oxidation of DCFH by ROS) in cell lysates was measured using a FLUOstar OPTIMA microplate fluorometer (BMG Lab Technologies, Durham, NC, USA). Absorbance values were expressed relative to protein content in cell lysates.

### 2.16. Statistical analysis

All results are expressed as means ± s.e.m., and differences were analysed for statistical significance using unpaired *t*-test, one-way analysis of variance (ANOVA) with Tukey's multiple comparison test or two-way ANOVA with Bonferroni post hoc test, as appropriate. Significance of differences is reported at *p* < 0.05, *p* < 0.01 and *p* < 0.001 levels, as indicated in figure legends.

## 3. Results

### 3.1. mRNA expression of hANT isoforms in HeLa cells under proliferative and cell-arrest conditions

**Figure 1.**
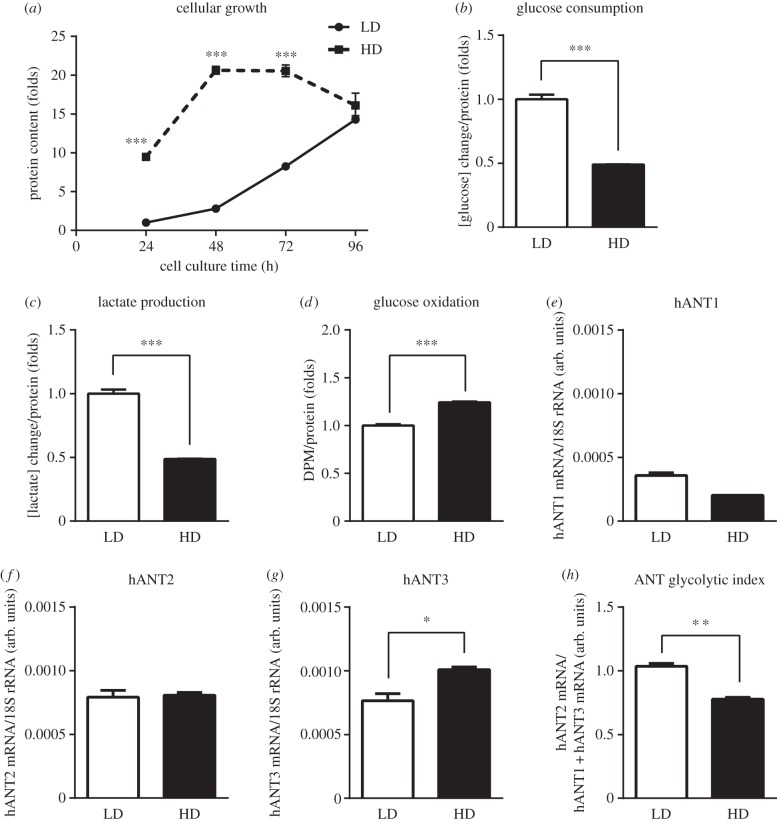
Non-proliferating HeLa cells show an increase in oxidative metabolism and hANT3 mRNA expression. (*a*) HeLa cells were plated at LD and HD, and cell growth was quantified every 24 h for 4 days by determining total protein in fixed cells using SRB assays. (*b*–*d*) Quantification of (*b*) glucose consumption, (*c*) lactate production and (*d*) glucose oxidation by HeLa cells under LD and HD conditions 48 h after seeding. (*e*–*h*) Determination of (*e*) hANT1, (*f*) hANT2 and (*g*) hANT3 mRNA levels by qPCR, and (*h*) calculation of the ANT glycolytic index in HeLa cells under LD and HD conditions 48 h after seeding. Values are means ± s.e.m. of three to six independent experiments. *p < 0.05, **p < 0.01 and ***p < 0.001 for the indicated comparisons.

To analyse the changes in hANT isoform mRNAs in cells stimulated to proliferate or undergo growth arrest, we plated HeLa cells at HD or LD and allowed them to grow for 96 h. As depicted in figure 1*a*, HD cells reached confluence after 48 h in culture, indicating that they were sensitive to cell contact-dependent growth arrest. By contrast, LD cells grew continuously until 96 h, at which point they reached the same confluent endpoint as HD cells. We chose 48 h of culture, when HD cells had stopped growing and LD cells were still proliferating, as the time for studying metabolic-dependent differences in the expression of hANT isoforms. At this time point, LD cells were more glycolytic, as evidenced by their greater consumption of glucose and production of lactate, and reduced rate of glucose oxidation compared with HD cells (figure 1*b*–*d*). HeLa cells expressed all three hANT isoforms; hANT2 and hANT3 were expressed at similar levels, whereas hANT1, the muscle-specific isoform, was expressed at lower levels (figure 1*e*–*g*). In growth-arrested (HD) cells, hANT1 expression decreased slightly, hANT2 did not change and hANT3 expression significantly increased compared with proliferating (LD) cells (figure 1*e*–*g*). Exploiting the distinct putative role of hANT2 in glycolytic metabolism compared with the function of hANT1 and hANT3 in oxidative metabolism, we developed an ANT glycolytic index, defined as the hANT2 mRNA/(hANT1 mRNA + hANT3 mRNA) ratio. We found that the ANT glycolytic ratio was lower in oxidative/growth-arrested (HD) cells than in glycolytic/proliferative (LD) cells (figure 1*h*). Interestingly, adaptive changes in the ANT glycolytic ratio were attributable to changes in the hANT3 isoform in HeLa cells, previously defined as ubiquitous, indicating that the regulation of this isoform is more important than previously assumed.

### 3.2. A decrease in the ANT glycolytic ratio is associated with a metabolic shift towards oxidation in HepG2 and SGBS cells

**Figure 2.**
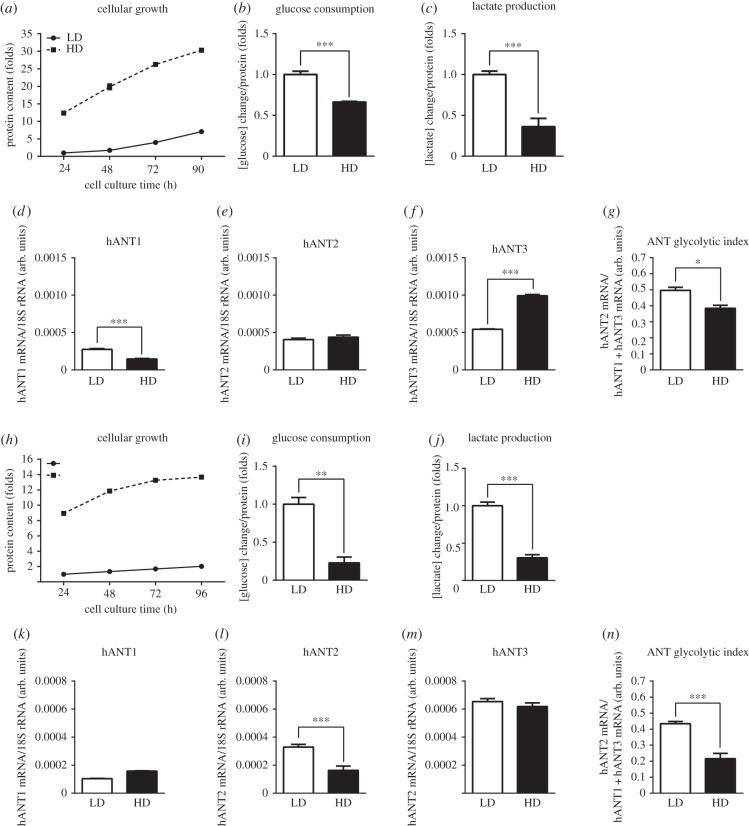
Cell growth arrest in HepG2 cells induces oxidative metabolism and hANT3 mRNA expression, whereas SGBS cell growth arrest is accompanied by a decrease in hANT2 mRNA levels with no change in hANT3 mRNA. (*a*) Quantification of HepG2 cells growth every 24 h for 4 days after seeding at LD and HD. (*b*–*c*) Quantification of (*b*) glucose consumption and (*c*) lactate production at 72 h in HepG2 cells plated under LD and HD conditions. (*d*–*g*) Determination of (*d*) hANT1, (*e*) hANT2 and (*f*) hANT3 mRNA levels by qPCR, and (*g*) calculation of the ANT glycolytic index in HepG2 cells 72 h after seeding. (*h*) Quantification of SGBS cell growth every 24 h for 4 days after seeding at LD and HD. (*i*–*j*) Quantification of (*i*) glucose consumption and (*j*) lactate production at 96 h in SGBS cells plated under LD and HD conditions. (*k*–*n*) Determination of (*k*) hANT1, (*l*) hANT2 and (*m*) hANT3 mRNA levels by qPCR, and (*n*) calculation of the ANT glycolytic index in SGBS cells 96 h after seeding. Values are means ± s.e.m. of three to six independent experiments. *p < 0.05, **p < 0.01 and ***p < 0.001 for the indicated comparisons.

To determine whether the regulation of hANT3 mRNA was restricted to HeLa cells, we expanded these studies to other human cell lines with or without susceptibility to cell-arrest regulation. To this end, we used HepG2 cells, a hepatocyte cell line derived from a human hepatocellular carcinoma that is not fully susceptible to cell contact-dependent cell arrest. We observed that HepG2 cells plated at HD and LD were able to continuously proliferate for at least 96 h (figure 2*a*). When cells were plated under HD conditions, their growth slowed but did not stop. In addition to the decrease in the rate of growth, cells plated under HD conditions showed a metabolic shift towards oxidation at 72 h after seeding, as indicated by a decrease in glucose uptake (figure 2*b*) and lactate production (figure 2*c*). These alterations in growth rate and the concomitant metabolic shift were accompanied by a marked increase in hANT3 mRNA expression (figure 2*f*), a small decrease in hANT1 (figure 2*d*) and no changes in hANT2 (figure 2*e*). Thus, hANT3 mRNA expression was increased in slow-growing HepG2 cells that had acquired an oxidative metabolism, resulting in a decrease in the ANT glycolytic index (figure 2*g*). We were also interested in studying the mRNA expression profile of ANT isoforms under proliferative and growth-arrest conditions in cells of a fibroblastic lineage, where hANT2 was first described as a cell proliferation marker [13]. For this purpose, we used SGBS cells, a human preadipocyte cell line fully susceptible to growth arrest [22]. SGBS cells grew at a slow rate even when plated at LD (figure 2*h*). At 96 h of culture, LD cells were still proliferating, whereas HD cells had already reached confluence and stopped growing. Similar to HeLa and HepG2 cells, SGBS cells under HD conditions also showed a decrease in glucose uptake (figure 2*i*) and lactate production (figure 2*j*). SGBS cells expressed approximately three times more hANT3 than hANT2 mRNA, and presented very low levels of hANT1 mRNA (figure 2*k*–*m*), a result in accord with the more oxidative metabolic profile of this preadipocyte cell compared with the highly proliferative, glycolytic HeLa and HepG2 cells. Cell growth arrest produced a marked decrease in hANT2 mRNA, with no change in hANT3 or hANT1 mRNA. This resulted in a decrease in the ANT glycolytic ratio (figure 2*n*), indicating a shift to a more oxidative metabolism upon growth arrest. In this case, however, the change in the ANT glycolytic ratio was attributable to changes in hANT2 isoform mRNA expression.

### 3.3. mRNA expression of hANT isoforms in HeLa cells under oxidative/glycolytic conditions

**Figure 3.**
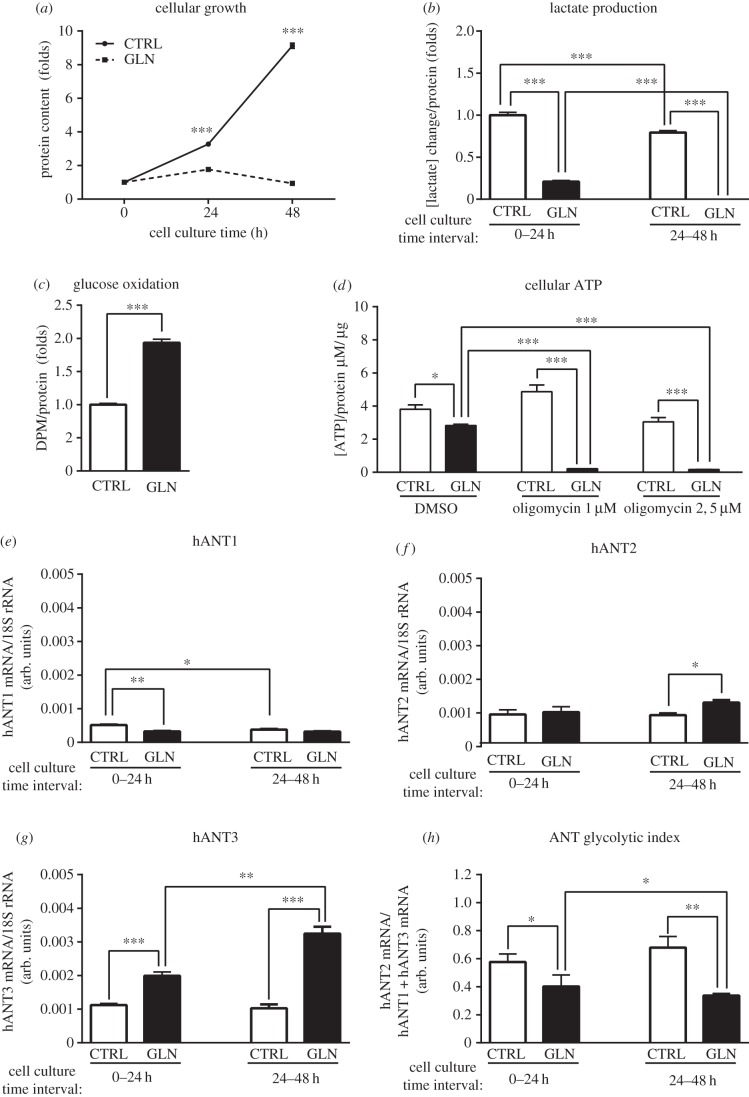
Suppression of glycolytic metabolism in HeLa cells triggers an induction of hANT3 mRNA expression. (*a*) Quantification of total protein content after 0, 24 and 48 h in HeLa cells maintained with complete medium (CTRL) or with glutamine medium (GLN) as a unique carbon source. (*b*–*d*) Quantification of (*b*) lactate production, (*c*) glucose oxidation and (*d*) cellular ATP in HeLa cells maintained in CTRL or GLN medium. (*e*–*h*) Determination of (*e*) hANT1, (*f*) hANT2 and (*g*) hANT3 mRNA levels, and (*h*) calculation of the ANT glycolytic index of HeLa cells incubated in CTRL or GLN medium. Values are means ± s.e.m. of three to six independent experiments. *p < 0.05, **p < 0.01 and ***p < 0.001 for the indicated comparisons.

To further study the relationship between the shift in glycolytic to oxidative metabolism and the expression of hANT isoforms, we incubated HeLa cells in media containing the non-fermentable substrate glutamine (GLN cells) without glucose or in complete medium (CTRL cells) containing both high glucose and glutamine [23,24]. The aim of this experiment was to force cells to acquire an oxidative metabolism (GLN cells) or shift metabolism towards glycolysis (CTRL cells). GLN cells incubated for 24 and 48 h stopped growing (figure 3*a*) and markedly reduced their production of lactate (figure 3*b*); when pulsed with glucose at 48 h of culture, they doubled their rate of glucose oxidation compared with CTRL cells (figure 3*c*), indicating a metabolic shift towards oxidative metabolism. The oxidative phenotype of GLN cells was further demonstrated by their strict dependence on OXPHOS for the maintenance of cellular ATP levels. As can be seen in figure 3*d*, CTRL cells maintained their ATP concentration in the presence of oligomycin, an ATP synthase inhibitor, indicating that they fulfilled their energy requirements mainly through glycolysis. By contrast, GLN cells were highly sensitive to oligomycin, indicating their dependence on OXPHOS. In this situation, hANT isoform mRNA levels changed accordingly: hANT1 and hANT2 mRNA showed only minor changes, whereas hANT3 mRNA markedly increased at both 24 and 48 h (figure 3*e*–*g*), resulting in a decrease in the ANT glycolytic ratio (figure 3*h*). Again, it was the expression of the hANT3 isoform that showed major changes.

### 3.4. Effects of growth factors and inhibition of growth-factor signalling on mRNA expression of hANT isoforms in HeLa cells

**Figure 4.**
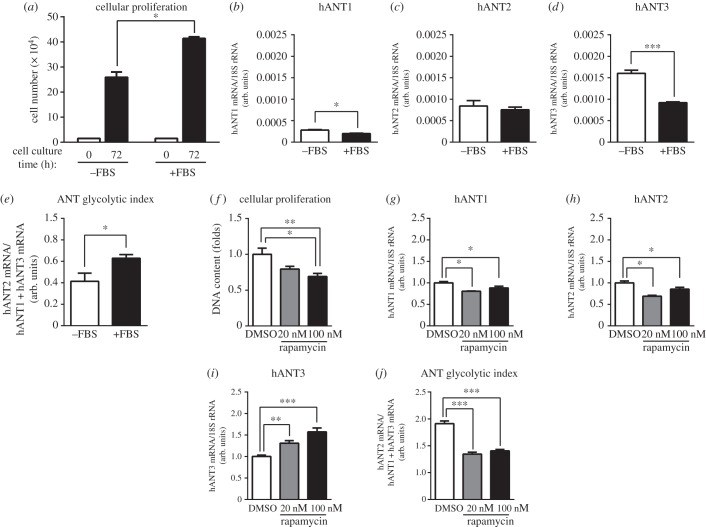
Inhibition of HeLa cell proliferation increases hANT3 mRNA levels. (*a*) Determination of HeLa cell proliferation by cell counting initially (0 h) and 72 h after maintaining cells in the absence (–FBS) or presence (+FBS) of FBS. (*b*–*e*) Determination of (*b*) hANT1, (*c*) hANT2 and (*d*) hANT3 mRNA levels, and (*e*) calculation of the ANT glycolytic index of HeLa cells incubated in the absence or the presence of FBS for 72 h. (*f*–*j*) Determination of (*f*) HeLa cell proliferation by quantification of DNA content; (*g*) hANT1, (*h*) hANT2 and (*i*) hANT3 mRNA levels; and (*j*) calculation of the ANT glycolytic index of HeLa cells incubated for 24 h with complete medium containing rapamycin (20 or 100 nM) or DMSO (vehicle). Values are means ± s.e.m. of three to six independent experiments. *p < 0.05, **p < 0.01 and ***p < 0.001 for the indicated comparisons.

Cell proliferation is dependent on growth factors. Accordingly, we sought to determine how growth factors affected the expression of hANT isoforms. To accomplish this, we incubated HeLa cells with or without FBS for 72 h. As shown in figure 4*a*, HeLa cells were only partially sensitive to FBS. Although they proliferated more in the presence of 10% FBS, they were nonetheless able to grow in the absence of FBS and thus were not fully susceptible to serum deprivation-induced growth arrest (figure 4*a*). FBS did not affect the expression of hANT2 or hANT1 mRNA (figure 4*b,c*), but did suppress hANT3 mRNA expression (figure 4*d*). Thus, growth factors present in FBS increased the ANT glycolytic ratio (figure 4*e*).

To gain insight into the regulation of hANT3 by a proliferative stimulus, we treated proliferating HeLa cells, plated at LD, with two different concentrations of rapamycin (20 or 100 nM), for 24 h. This compound is a known inhibitor of mTOR, a factor that lies at a crucial point in the integration of molecular signals induced by growth factors [25]. Inhibition of mTOR by rapamycin decreased cell proliferation (figure 4*f*), slightly downregulated both hANT1 and hANT2 expression, an effect that showed inconsistent concentration dependence, but substantially increased the expression of hANT3 mRNA (figure 4*g*–*i*), resulting in a decrease in the ANT glycolytic ratio (figure 4*j*). However, the possibility that the changes in hANT3 mRNA levels induced by rapamycin treatment were attributable to factors other than inhibition of cell proliferation cannot be discounted.

### 3.5. Overexpression of hANT2 induces cell growth and glycolytic metabolism

**Figure 5.**
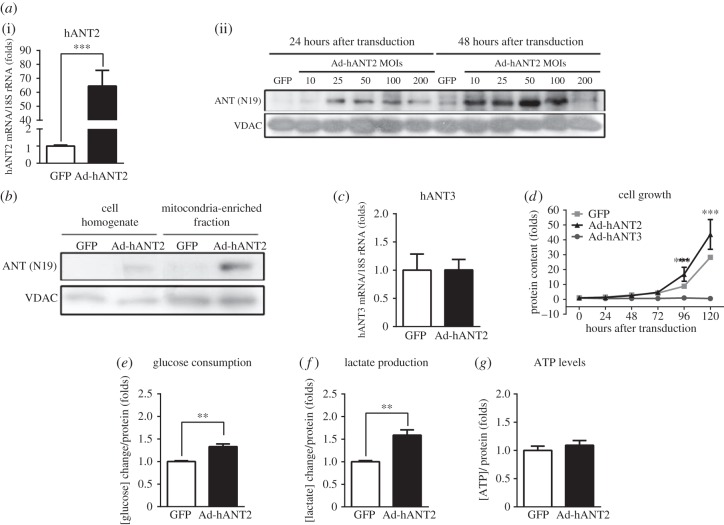
hANT2 overexpression promotes proliferation and induces glycolytic metabolism in HeLa cells. (*a*) Expression of hANT2 in HeLa cells, determined by qPCR 48 h after adenoviral transduction of Ad-GFP or Ad-hANT2 at a MOI of 50 (i), and by Western blot analysis 24 or 48 h after adenoviral transduction of Ad-GFP or Ad-hANT2 at several different MOIs (ii). (*b*) Western blot analysis showing the expression of ANT in total cell homogenates or mitochondria-enriched fractions of HeLa cells 48 h after adenoviral transduction of Ad-GFP or Ad-hANT2. (*c*) Quantification of hANT3 expression determined by qPCR in HeLa cells 48 h after adenoviral transduction of Ad-GFP or Ad-hANT2 at a MOI of 50. (*d*) Analysis of the growth of hANT2 or hANT3 isoform-overexpressing HeLa cells by quantification of protein content by SRB staining every 24 h. (*e*–*g*) Quantification of (*e*) glucose consumption, (*f*) lactate production and (*g*) ATP levels in HeLa cells 48 h after transducing with Ad-GFP or Ad-hANT2 at an MOI of 50. Values are means ± s.e.m. of four to six independent experiments. **p < 0.01 and ***p < 0.001 for the indicated comparisons.

Having established that the expression of distinct hANT isoforms is regulated in a manner that depends on the metabolic and/or proliferative status of the cells, we sought to investigate the proliferative and metabolic consequences triggered by changing the relative expression of hANT2 and hANT3 isoforms. We first addressed this issue by overexpressing hANT2 and hANT3 in HeLa cells. Adenoviral transduction at an MOI of 50 for 48 h was sufficient to obtain adequate hANT2 overexpression compared with GFP transduced cells (transduction control), as determined by mRNA and protein expression (figure 5*a*). We also validated that overexpressed hANT2 protein was localized to mitochondria (figure 5*b*) and that overexpression of hANT2 did not affect the expression of hANT3 mRNA (figure 5*c*). Overexpression of hANT2 in HeLa cells using these optimized transduction conditions increased cell growth at 96 h after transduction (figure 5*d*). HeLa cells overexpressing hANT2 also exhibited increased glucose consumption (figure 5*e*) and lactate production (figure 5*f*), indicating enhanced glycolytic metabolism. Under these conditions, ATP levels were not affected (figure 5*g*), suggesting that this change in glucose metabolism did not compromise the energy status of HeLa cells. On the contrary, overexpression of hANT3 stopped cell growth, in accordance with our previous observation that hANT3 overexpression in cultured cells promotes apoptosis [20]. Overexpression of hANT2 in HepG2 cells also triggered a shift from oxidative to glycolytic metabolism (data not shown).

### 3.6. Silencing of hANT2 and hANT3 isoforms

**Figure 6.**
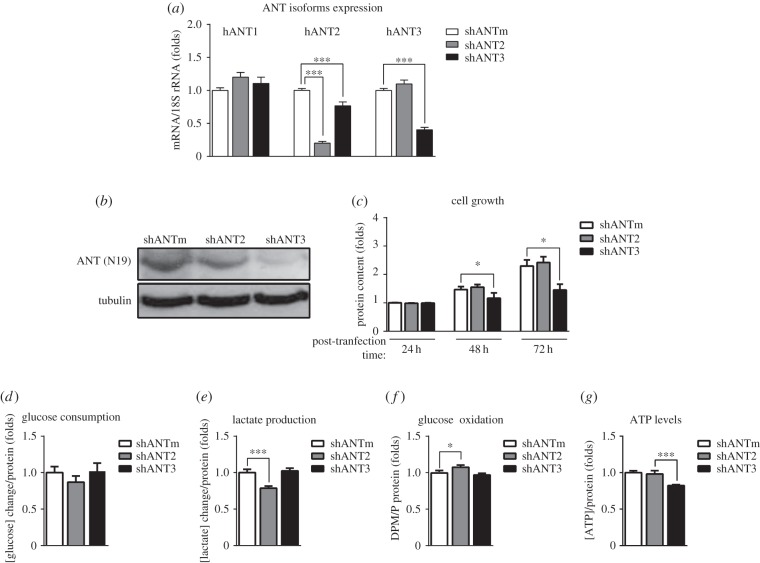
Changes in hANT isoforms expression determine glucose metabolism. Quantification of the expression of hANT1–3 isoforms by (*a*) qPCR or (*b*) Western blot analysis in control (shANTm) HeLa cells and hANT2-knockdown (shANT2) and ANT3-knockdown (shANT3) cells 48 h after shRNA transfection. (*c*) Analysis of cell growth by quantification of protein content by SRB staining every 24 h in shANTm, shANT2, and shANT3 HeLa cells. (*d*–*g*) Quantification of (*d*) glucose consumption, (*e*) lactate production, (*f*) glucose oxidation and (*g*) ATP levels in shANTm, shANT2 and shANT3 HeLa cells 48 h after shRNA transfection. Values are means ± s.e.m. of six to eight independent experiments. *p < 0.05 and ***p < 0.001 for the indicated comparisons.

As a complement to hANT2-overexpression approaches, we also studied the effects of changing the relative expression of hANT2 and hANT3 in HeLa cells by isoform-specific silencing using shRNA-mediated knockdown to better understand the role of specific ANT isoforms in metabolism. As shown in figure 6*a,b*, isoform-specific shRNAs effectively silenced hANT2 and hANT3 at the mRNA level, resulting in a concomitant reduction in hANT3 protein, but only a modest decrease in hANT2 protein. hANT2 knockdown did not affect cell growth (figure 6*c*), most probably because of its minimal effects on protein expression. However, hANT2 silencing did reduce lactate production and increase glucose oxidation (figure 6*d–f*), demonstrating a direct role of the hANT2 isoform in regulating oxidative metabolism. This metabolic adaptation did not change the energy status of cells, as indicated by the maintenance of ATP levels (figure 6*g*). On the other hand, cells with low levels of hANT3 expression completely stopped cell growth (figure 6*c*) and were unable to shift their metabolism towards a more glycolytic state (figure 6*d*–*f*), resulting in a significant decrease in intracellular ATP levels indicative of an impaired energy status (figure 6*g*). It is likely that this lack of sufficient ATP is responsible for preventing cell growth. These results demonstrate that hANT3 is essential in slow-proliferating HeLa cells.

### 3.7. Silencing of hANT3 induces a cell stress response

**Figure 7.**
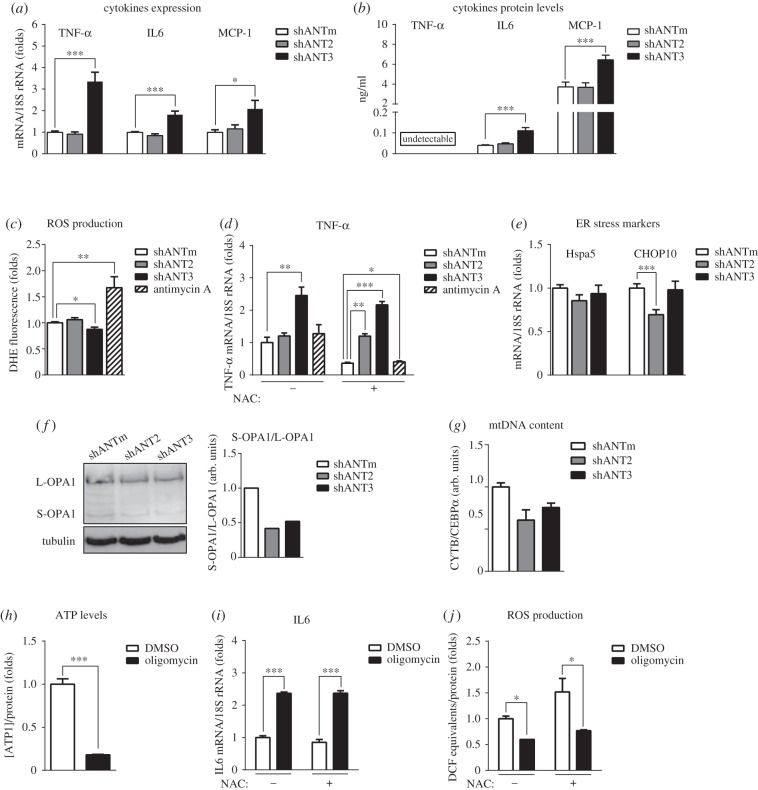
hANT3 knockdown induces the expression of cytokines. Quantification of TNF-α, IL6 and MCP-1 cytokine (*a*) mRNA levels and (*b*) protein in media, (*c*) ROS production, (*d*) TNF-α in the presence or the absence of NAC by qPCR, and (*e*) expression of ER stress markers HSPA5 and CHOP10 by qPCR in control (shANTm), hANT2-knockdown (shANT2) and hANT3-knockdown (shANT3) HeLa cells 48 h after shRNA transfection. Antimycin A treatment served as a positive control for ROS production. (*f*) Immunodetection of OPA1 by Western blot analysis, and (*g*) quantification of mtDNA content in shANTm, shANT2 and shANT3 HeLa cells 48 h after shRNA transfection. (*h*–*j*) Quantification of (*h*) ATP levels in HeLa cells treated with 10 µM oligomycin A for 30 min, (*i*) IL6 expression determined by qPCR and (*j*) ROS production in HeLa cells treated with 10 µM oligomycin A for 3 h in the presence or the absence of 10 mM NAC. Values are means ± s.e.m. of three independent experiments. *p < 0.05, **p < 0.01 and ***p < 0.001.

The inability of HeLa cells to compensate for hANT3 silencing at the energy level prompted us to investigate how cells sensed the decrease in hANT3 function. To this end, we studied cytokine production as an indicator of cell stress response [26]. Silencing of hANT2 had no effect on the expression of cytokines; importantly, hANT3 knockdown significantly induced the expression of TNF-α, IL6 and MCP-1 in HeLa cells (figure 7*a*). This induction in mRNA levels correlated with an increase in the protein levels of IL6 and MCP-1 in cell culture media (figure 7*b*). However, the levels of TNF-*α* protein were below the lower limits of detection of the method used. shRNA-mediated hANT3 knockdown did not result in an increase in the levels of ROS (figure 7*c*), known intermediates in the cytokine production response [27], whereas antimycin A, a known ROS inducer [28], did induce ROS production, indicating the functionality of this pathway in HeLa cells. Moreover, pretreatment with the antioxidant NAC did not suppress the induction of TNF-α expression in hANT3-knockdown HeLa cells (figure 7*d*), as NAC did with the cells treated with antimycin A. Collectively, these results indicate that ROS did not mediate the stress response associated with hANT3 knockdown in HeLa cells. Another recognized intermediate in the stress response is ER stress [29]. The induction of cytokine expression by hANT3 silencing also did not appear to involve an ER stress response, as evidenced by the lack of changes in expression of the ER stress markers HSPA5 and CHOP10 under these conditions (figure 7*e*). Finally, we investigated the possible participation of mitochondrial stress as an inducer of cellular stress [30]. First, we analysed the levels of OPA1, a protein involved in mitochondrial fusion. Under mitochondrial stress situations, the long form of OPA1 (L-OPA1) is totally proteolysed to the short form (S-OPA1), impairing mitochondrial fusion and inducing mitochondrial fragmentation [31]. hANT3-knockdown HeLa cells showed no accumulation of the stress-induced S-OPA1 form, which if anything trended lower (figure 7*f*). Likewise, mitochondrial stress has also been associated with an increase in mtDNA content [32]. Neither hANT2 nor hANT3 knockdown in HeLa cells produced an increase in mtDNA content, instead slightly decreasing it (figure 7*g*). Thus, hANT3 silencing seemed not to result in significant mitochondrial stress. Furthermore, neither hANT2 nor hANT3 silencing triggered significant changes in mitochondrial membrane potential (data not shown).

Having eliminated a role for ROS production, ER stress and mitochondrial stress in the stress response triggered by hANT3 silencing, we considered the possible involvement of the decrease in ATP levels. To test this, we treated confluent HeLa cells with 10 µM oligomycin, an ATP synthase inhibitor. As expected, since in these conditions HeLa cells are oxidative (figure 1), oligomycin created an energy failure situation, as demonstrated by a dramatic decrease in ATP levels (figure 7*h*). Interestingly, the decrease in ATP levels was accompanied by an increase in IL6 expression (figure 7*i*). This effect was completely independent of ROS production, as demonstrated by the failure of NAC to alter oligomycin-induced IL6 gene expression and the fact that ROS levels were even lower in oligomycin-treated HeLa cells (figure 7*j*). Collectively, these results suggest that the induction of cytokine production by a decrease in hANT3 is not mediated by an increase in ROS production. Hence, it is reasonable to conclude that the cell stress response induced by hANT3 silencing could be induced by a decrease in the energy status of cells.

## 4. Discussion

The principal finding of this study is that hANT3 is the main regulated hANT isoform in HeLa and HepG2 cells, which are not fully sensitive to growth factor deprivation or inhibition of growth by cell contact, respectively. hANT3 was upregulated by the induction of growth arrest as well as by forced acquisition of an oxidative metabolism. Whether hANT3 regulation is related specifically to one of these two processes is not known, but both processes—proliferation and glycolytic metabolism—appear to be intimately and mechanistically related to each another [33]. By contrast, in SGBS preadipocytes, which are fully susceptible to growth arrest, hANT3 expression remained stable; instead, it was the hANT2 isoform that became growth-regulated. It has long been recognized that hANT2 expression is highly regulated. hANT2 was first cloned as an early response gene in human diploid fibroblasts [10] and is considered a marker of cell proliferation [13]. The promoter of the *ANT2* gene has been extensively studied, mainly by Nelson and colleagues, who identified the promoter regions responsible for gene silencing in quiescence [34], and also by Stepien and co-workers, who described a GRBOX in the *ANT2* gene promoter that is involved in linking high glycolysis to upregulation of this gene [14]. Our results showed that this sensitivity of hANT2 to glycolysis and proliferation is not a general phenomenon, but instead is cell specific. Unlike the relatively well-defined regulatory features of the h*ANT2* gene, there is scant knowledge about the regulation of hANT3 expression. Typical of the so-called housekeeping genes, the promoter region of the h*ANT3* gene lacks TATA and CAT boxes, and has multiple SP1-binding sites [35], presumably dampening enthusiasm for more extensive studies. Additionally, a recent bioinformatics study on the regulation of hANT isoforms expression that compared common characteristics of the proximal promoter regions of other genes with those in each of the human ANT isoforms failed to detect a pattern of genes that were co-regulated with hANT3, a result in line with the ubiquitous expression of this isoform and apparent absence of a requirement for specific regulation [36]. Nevertheless, our results suggest that the expression of hANT3 may be as highly regulated as that of hANT2. Interestingly, the regulation of hANT2 and hANT3 expression was mutually exclusive and highly cell-type-dependent.

The fact that all species tested to date, from yeast to mammals, have several ANT isoforms with specific regulated expression and tissue-specific expression patterns [6–8] strongly suggests specific roles and/or properties of the different isoforms. Several lines of evidence support this conclusion. For example, hANT1 expression is restricted to tissues with high OXPHOS activity, like heart, muscle and brain, whereas hANT3 is the main hANT isoform in oxidative non-muscle tissues. Accordingly, hANT1 and hANT3 appear to be directly involved in OXPHOS, acting as classic ATP/ADP translocases to export newly produced ATP synthetized by mitochondrial ATP synthase and import ADP from the cytosol to facilitate its rephosphorylation. Our demonstration that hANT3 expression is upregulated in HeLa and HepG2 cells upon a metabolic shift to oxidation highlights the oxidative role of this isoform. By contrast, hANT2 is induced in tissues with a high regenerative capacity (e.g. liver) and in proliferating cells (e.g. embryonic, blood cells and cancer cells) [6–8,10] characterized by low OXPHOS activity and high glycolysis, even in the presence of oxygen (Warburg effect) [37]. This expression pattern points to a special role for this isoform in the context of OXPHOS. Specifically, it has been proposed that hANT2 may preferentially exhibit reverse ATP/ADP activity that would fuel mitochondria with ATP produced by glycolysis [14].

Taking into account the opposite roles of ANT isoforms in oxidative/glycolytic metabolism, we considered that a ratio of the expression of hANT isoforms would be more closely related to cell conditions than the expression of individual hANT isoforms. Hence, we proposed an index, the ANT glycolytic index, based on mRNA levels of glycolytic ANT isoform divided by the mRNA levels of oxidative isoforms (hANT2 mRNA/(hANT1 mRNA + hANT3 mRNA)). Our goal was to determine whether changes in this ratio were a leading indicator of glycolytic/oxidative status (or the closely related proliferative/growth-arrest status) of cells and thus could be used as a facile method for predicting cell status. Our results have clearly shown that this index did accurately reflect changes in the proliferation/quiescence and glycolytic/oxidative status of cells regardless of the specific hANT isoform changing—hANT2 in some cases, but hANT3 in others. In any case, the meaning of the ANT glycolytic index remains the same: it increases when cells proliferate and shift their metabolism towards glycolysis, and decreases when cells stop growing and shift their metabolism towards oxidation. Thus, by calculating this index under different cellular conditions, it should be possible to predict changes in the metabolic/proliferative status of cells. Nonetheless, further analysis in a wider spectrum of human cell lines should be done to validate this index.

We also studied the metabolic consequences of manipulating the expression of hANT2 and hANT3 isoforms. Our results indicate that hANT2 overexpression in HeLa cells promotes glycolysis and also increases cell growth, findings in agreement with the previous observation that glycolytic metabolism can induce proliferation [38], and with the original description of hANT2 as an early response gene and proliferation marker [10]. The fact that hANT2 overexpression enhances the classic cancer cell phenotype in HeLa cells, which, like the majority of tumour-derived cells, have basal high levels of hANT2 expression and a predominant glycolytic metabolism, reveals the importance of hANT2 in regulating cellular metabolism. Thus, our data from overexpression studies demonstrate the ability of the hANT2 isoform *per se* to induce the shift from oxidative to glycolytic metabolism and promote cell growth. It has been shown that hANT2 silencing is a successful strategy for reducing breast cancer cell viability [19]. In our study, the lack of clear effects of hANT2 knockdown on growth rate could be explained by an insufficient reduction of hANT2 protein, despite effective knockdown at the mRNA level. However, hANT2 silencing and the consequent decrease in the glycolytic index did induce oxidative metabolism.

Overexpression of hANT3 in cultured cells precludes the study of metabolic consequences of changes in h*ANT3* gene expression owing to the proapoptotic effects of this isoform [20]. Nevertheless, we were able to study hANT3 function by silencing its expression. hANT3 knockdown markedly slowed cell growth, an effect that seemed to be related to a decrease in cellular ATP levels, owing to the inability of hANT3-silenced HeLa cells to compensate this energetic failure by increasing the glycolytic metabolism. The simplest explanation for the impairment in ATP levels by hANT3 silencing is that hANT3 is essential for proper OXPHOS activity. The results in HeLa cells are very surprising because cancer cells, such as HeLa cells, are characterized by a very low oxidative metabolism; thus, *a priori* they would not have been expected to be dependent on OXPHOS and therefore on hANT3 activity. But, as noted above, under our experimental conditions, cells were near confluence and therefore more dependent on hANT3. Collectively, our results seem to indicate that cancer cells are dependent on hANT2 when proliferating at high rates and on hANT3 when proliferation slows.

In addition to its metabolic and proliferative consequences, hANT3 silencing induced the transcription of genes encoding the cytokines TNF-*α*, IL6 and MCP-1, a response that is indicative of the intense cellular perturbation produced by the decrease in hANT3 expression. One possible explanation for activation of an inflammatory response—an increase in ROS production due to impaired mitochondrial activity [39,40]—would seem to be ruled out by our demonstration that there was no relationship between hANT3 knockdown-induced cytokine expression and ROS production. Instead, the decrease in ATP levels and induction of IL6 expression by the ATP synthase inhibitor oligomycin suggest a direct relationship between energy impairment by hANT3 silencing and the induction of cytokine expression.

In summary, the mRNA expression of hANT isoforms is differentially regulated in a cell- and condition-dependent manner. In cells that are not fully responsive to growth arrest, such as HeLa and HepG2 cells, expression of hANT3 mRNA was upregulated upon a shift in cellular metabolism towards oxidation or cessation of cell growth. By contrast, in cells fully capable of growth arrest, like SGBS preadipocytes, hANT2 mRNA expression was downregulated under these same conditions. The ANT glycolytic index closely correlated with the metabolic/proliferative status of cells independent of the hANT isoform actually undergoing regulation and could be used as a predictor of the metabolic/proliferative status of cells. Moreover, hANT3 is essential for cell growth and its silencing results in energy impairment and a cell stress-like response. On the other hand, hANT2 by itself is able to induce cell proliferation and shift cell metabolism towards glycolysis altogether. Thus, cancer cells require both hANT2 and hANT3 depending on their proliferation status: hANT2 when cells proliferate at high rates and hANT3 when proliferation slows.
